# Adjunctive value of 3D MRCP in biliary atresia: a retrospective two-center analysis of cholestatic infants

**DOI:** 10.1186/s13244-025-02165-5

**Published:** 2025-12-17

**Authors:** Shuyi Liu, Rui Zhang, Yuqin He, Yuyun Liu, Rui Wang, Zidong Zhou, Hongbin Ma, Xialing He, Simin Yan, Li Huang, Kuiming Jiang, Hongsheng Liu

**Affiliations:** 1 Department of Radiology, Guangzhou Women and Children’s Medical Center, Guangzhou Medical University, Guangzhou, China; 2 Department of Ultrasound, Guangzhou Women and Children’s Medical Center, Guangzhou Medical University, Guangzhou, China; 3 Department of Radiology, Guangdong Women and Children Hospital, Guangzhou, China; 4 Jinan University, Guangzhou, China; 5 School of Pediatrics, Guangzhou Medical University, Guangzhou, China; 6 Department of Radiology, The Sixth Affiliated Hospital of Jinan University, Jinan University, Dongguan, China

**Keywords:** Biliary atresia, Cholestasis, Magnetic resonance imaging, Ultrasound

## Abstract

**Objectives:**

To evaluate the adjunctive diagnostic value of three-dimensional MR cholangiopancreatography (3D MRCP) for identifying biliary atresia (BA) in infants with cholestasis.

**Materials and methods:**

This retrospective two-center study evaluated the adjunctive diagnostic performance of 3D MRCP beyond ultrasound (US) using receiver operating characteristic (ROC) analysis. The cohort from center 1 was divided into training (*n* = 770) and validation (*n* = 330) sets, with center 2 as the test set (*n* = 252). The optimal cut-off for the MR triangular cord thickness (MR-TCT) was derived from the area under the ROC curve (AUC) calculated from all cases. Extrahepatic bile ducts visualization on 3D MRCP was validated against surgical findings. Image quality metrics were assessed for their diagnostic value on BA detection.

**Results:**

One thousand three hundred fifty-two eligible cholestatic infants undergoing 3D MRCP (February 2012 to June 2023) were enrolled, including 363 BA and 989 non-BA. ROC analysis identified 3.75 mm as the optimal cut-off MR-TCT for BA diagnosis (AUC = 0.828). The combination of MR-TCT, 3D MRCP, and US yielded superior diagnostic performance, achieving AUCs of 0.967 in the training set, 0.958 in the validation set, and 0.972 in the test set (all *p* < 0.001). Image quality scores (*p* < 0.001), signal-to-noise ratio (SNR) (*p* < 0.001), contrast ratio (*p* = 0.012), and contrast-to-noise ratio (CNR) (*p* < 0.001) of 3D MRCP significantly differed between correct and incorrect diagnosis groups.

**Conclusions:**

3D MRCP is a valuable diagnostic adjunct tool in diagnosing BA, particularly when combined with MR-TCT and US. Optimizing 3D MRCP image quality enhances diagnostic accuracy.

**Critical relevance statement:**

3D MRCP enhances BA diagnosis when combined with MR-TCT and US. Importantly, in cases with strong clinical suspicion but negative US findings, MRCP should be utilized as an adjunct diagnostic modality to reduce false-negative rates.

**Key Points:**

- The diagnostic efficacy of 3D-MRCP in BA remains to be fully characterized.
- MR-TCT, 3D-MRCP, and US combined achieved optimal diagnostic accuracy for BA.
- For high-suspicion BA with negative US, adjunct 3D-MRCP reduces false-negative diagnoses.

**Graphical Abstract:**

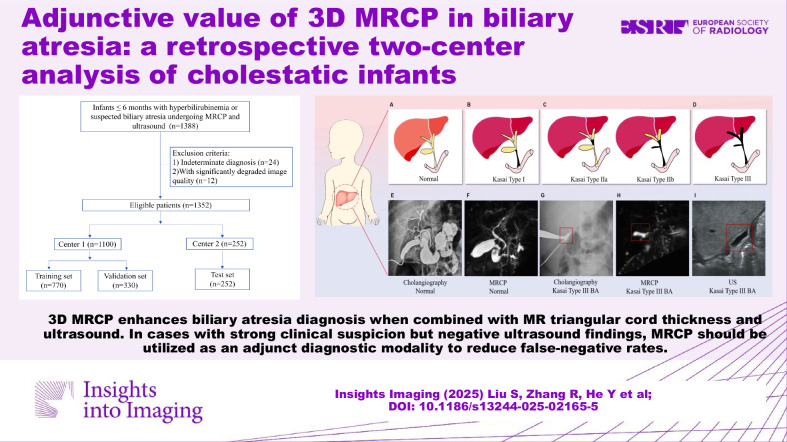

## Introduction

Biliary atresia (BA) is a rare yet progressive obliterative cholangiopathy that leads to the fibrosis and eventual destruction of the intrahepatic and extrahepatic bile ducts [1–3]. Although its exact etiology remains unknown, BA is the leading cause of neonatal cholestasis, responsible for 25–35% of cases [4]. Early identification and accurate differentiation of BA from other causes of neonatal jaundice are crucial, as the prognosis of BA is closely tied to the timing of surgical intervention. The Kasai portoenterostomy, a procedure designed to restore bile flow by bypassing the obstructed bile ducts, offers the best chance for long-term survival [5]. However, the success of this operation is significantly higher when performed in infants younger than 90 days of age, emphasizing the importance of early diagnosis [6].

Several imaging modalities are used to diagnose BA, with ultrasonography (US) being one of the most commonly employed techniques due to its convenience and cost-effectiveness. Characteristic US findings, such as the triangular cord sign, absent extrahepatic bile ducts, an enlarged hepatic artery, subcapsular hepatic flow, and the “gallbladder ghost triad” (atrophic gallbladder, irregular gallbladder contour, and interrupted gallbladder mucosa), can suggest BA [7–9]. However, the accuracy of US is limited by its operator dependency, which can pose challenges in settings with less experienced practitioners. In contrast, three-dimensional magnetic resonance cholangiopancreatography (3D MRCP) provides high-resolution visualization of both intrahepatic and extrahepatic bile ducts. 3D MRCP allows for more consistent and clearer detection of biliary anomalies, proving especially valuable in complex or diagnostically challenging cases [10, 11]. While previous studies have been limited by small sample sizes and single-center designs, the diagnostic utility of MRCP in BA remains an area of ongoing investigation [12–14].

The study aimed to assess the adjunctive value of 3D MRCP beyond US in diagnosing BA among cholestatic infants. Furthermore, it sought to determine the MR-TCT cut-off, validate 3D MRCP findings surgically, and evaluate the impact of image quality on diagnostic performance.

## Materials and methods

### Participants and baseline characteristics

**Fig. 1:**
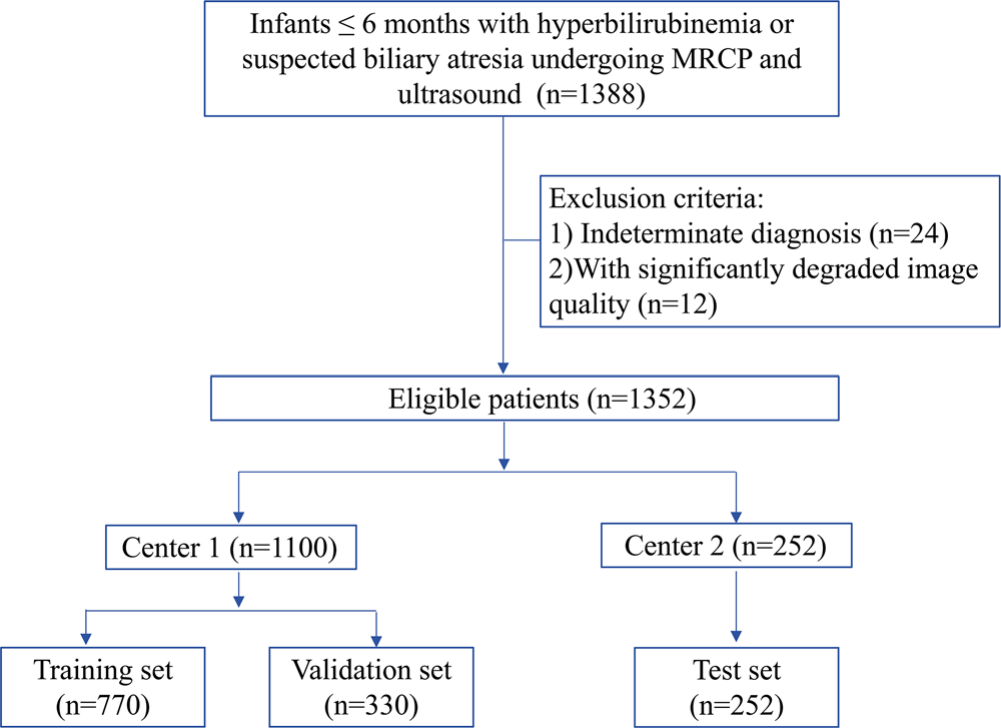
Flowchart of study participant selection for the training, validation, and test sets

This retrospective study received ethical approval from the Ethics Committee of Guangzhou Women and Children’s Medical Center (approval no. 2024-002B00) on March 15, 2024. Written informed consent was waived by the Institutional Review Board due to the retrospective nature of the study. All eligible patients identified between February 2012 and June 2023 were enrolled. Inclusion criteria were as follows: (a) infants were diagnosed with hyperbilirubinemia (with a serum direct bilirubin level > 17.1 μmol/L and a direct-to-total bilirubin ratio > 20%) or suspected of having BA, (b) ≤ 6 months of age, and (c) underwent both abdominal 3D MRCP and US. Exclusion criteria: (a) with an indeterminate diagnosis and (b) with significantly degraded image quality due to motion artifacts (Fig. 1).

### MRI acquisition and interpretation

The detailed MRI acquisition parameters were provided in Supplementary Table S1. 3D MRCP acquisitions utilized a respiratory-triggered or navigator-gated 3D fast spin-echo pulse sequence, obtaining isotropic or near-isotropic resolution. Patients fasted overnight for a minimum of 4 h prior to the examination. The acquisition time for the optimized 3D MRCP sequence used in the study ranged from approximately 3–5 min, depending on the infants’ respiratory rate and depth. Infants were sedated prior to the MRI examination with oral chloral hydrate, administered at a dose of 0.5 mL/kg. The failure to visualize any segment of the extrahepatic biliary tree (including the right/left hepatic ducts, common hepatic duct, and common bile duct) served as a definitive diagnostic criterion for BA on 3D MRCP [15]. All MRI images were reviewed by a radiologist (S.L.) with 10 years of experience in abdominal MRI. The radiologist was blinded to the clinical findings throughout the procedure.

### US acquisition and interpretation

All patients underwent standardized fasting for 4 h prior to the US examination. The US diagnosis of BA was based on a comprehensive evaluation of the positive imaging features, including: visibility of the common bile duct, GB morphology, GB contractility, US-TCT, main hepatic artery and portal vein diameters, presence or absence of hepatic subcapsular flow on Doppler US [7–9]. GB contractility was evaluated via pre-/post-feeding US with a 30-min interval.

### Qualitative and quantitative image analysis

**Fig. 2:**
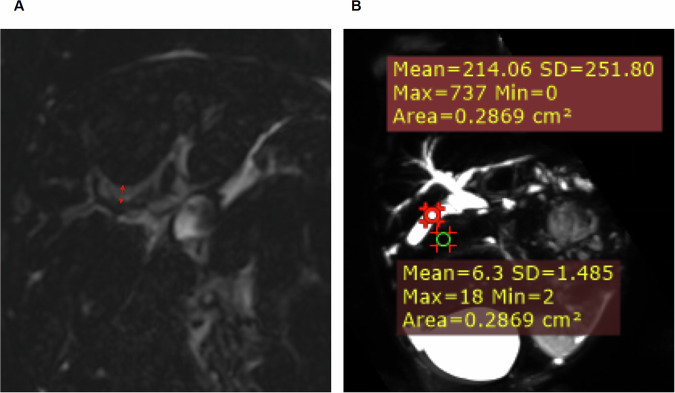
**A** MR-TCT was measured at the hyperintense SI localized anterior to the portal vein on 3D MRCP. **B** Standard region of interest placements for the GB (red circle), and the surrounding tissue (green circle) on 3D MRCP

The visualization of the right and left hepatic ducts, common hepatic ducts, and common bile duct was evaluated. 3D MRCP image quality was assessed using a five-point Likert scale as follows: 5 = excellent image quality, fully interpretable with no artifacts; 4 = good image quality, interpretable with minimal artifacts; 3 = average image quality, mildly affected by artifacts; 2 = below average image quality, interpretable but moderately affected by artifacts; 1 = poor image quality, uninterpretable. The triangular cord thickness (TCT) was quantitatively measured on coronal 3D MRCP images. This measurement was defined as the maximal anteroposterior dimension of the hyperintense triangular structure situated anterior to the bifurcation, specifically that of the anterior branch of the right portal vein (Fig. 2A). Signal intensity (SI) and image noise measurements were performed on the best-visualized and maximal gallbladder (GB) center and perigallbladder tissue. Image noise was defined as the mean standard deviation (SD) within each ROI. A homogeneous, artifact-free area of at least 5 mm² was placed in the GB and perigallbladder tissue (Fig. 2B). The signal-to-noise ratio (SNR), contrast ratio, and contrast-to-noise ratio (CNR) were calculated on 3D MRCP as follows:

$${{\rm{SNR}}} 	 = {meanSI}_{GB}/meanSD_{GB} \\ {{\rm{Contrast}}} \, {{\rm{ratio}}} 	 = \frac{({meanSI}_{GB}-{meanSI}_{{peripheral} \, {tissue}})}{({meanSI}_{GB} + {meanSI}_{peripheral \, tissue})} \\ {{\rm{CNR}}} 	 = \frac{{meanSI}_{GB}-{meanSI}_{peripheral tissue}}{\sqrt{({meanSD}_{GB}^{2} + {meanSD}_{{peripheral}}^{2})}/2}$$

For intra/inter-rater reliability, three radiologists participated in the image evaluation within a randomly selected subset of 100 patients. One radiologist (S.L.) performed the readings twice with an interval of at least two weeks to assess intra-rater reliability. For inter-rater reliability, two additional radiologists independently assessed the images: R.W. evaluated 3D MRCP quality (image quality score, SNR, contrast ratio, and CNR) and measured MR-TCT values, and Z.Z. analyzed bile duct visibility to confirm or exclude BA.

### Surgical exploration

Diagnoses were confirmed via intraoperative cholangiography during laparoscopy, laparoscopic exploration, liver biopsy, and/or follow-up. For those who underwent exploratory surgery, the development of the extrahepatic biliary duct was assessed based on intraoperative cholangiography and laparoscopic exploration. Patients were then classified into the BA group and the non-BA group.

### Statistical analysis

To analyze the data, SPSS version 26, MedCalc version 20, and Python version 3.12.9 (https://www.python.org) were used. The Shapiro–Wilk test was applied to assess the normality of the data. Data that followed a normal distribution were expressed as the mean ± SD, and *t*-tests were conducted to compare differences between the BA group and the non-BA group. For data that did not follow a normal distribution, the median with interquartile range (IQR) was reported, and the Mann–Whitney U test was used for group comparisons. The diagnostic performance of 3D MRCP for bile duct visualization was evaluated in terms of sensitivity, specificity, positive predictive value (PPV), negative predictive value (NPV), and accuracy. Also, the performance of various models was assessed using receiver operating characteristic (ROC) curves with area under the ROC curve (AUC) values. The DeLong test was employed to compare the differences in ROC curves between the models. Inter-reader and intra-reader agreements were assessed via Kappa, ICC, and Bland–Altman plots. A Kappa or ICC ≥ 0.75 denotes good reliability. A *p* value of ˂ 0.05 was regarded as statistically significant.

## Results

### Patient characteristics

The study enrolled 1352 infants from two centers (1100 from center 1 and 252 from center 2), including 363 BA (26.8%) and 989 non-BA (73.2%). The cohort was divided into training (*n* = 770) and validation (*n* = 330) sets from center 1 in a 7:3 ratio, and a test set (*n* = 252) from center 2. Age and gender distribution differed significantly between the BA and non-BA groups (both *p* < 0.001; Table 1).

**Table 1:** Baseline characteristics of the two groups

Characteristic	BA group (*n* = 363)	Non-BA group (*n* = 989)	*p* value	χ²/Z value
Sex (%)			< 0.001	32.442
Female	173 (47.7%)	306 (30.9%)		
Male	190 (52.3%)	683 (69.1%)		
Age at MR, median days (interquartile range)	58 (39, 68)	66 (50, 85)	< 0.001	−8.044

### Diagnostic performance of multimodal models

**Fig. 3:**
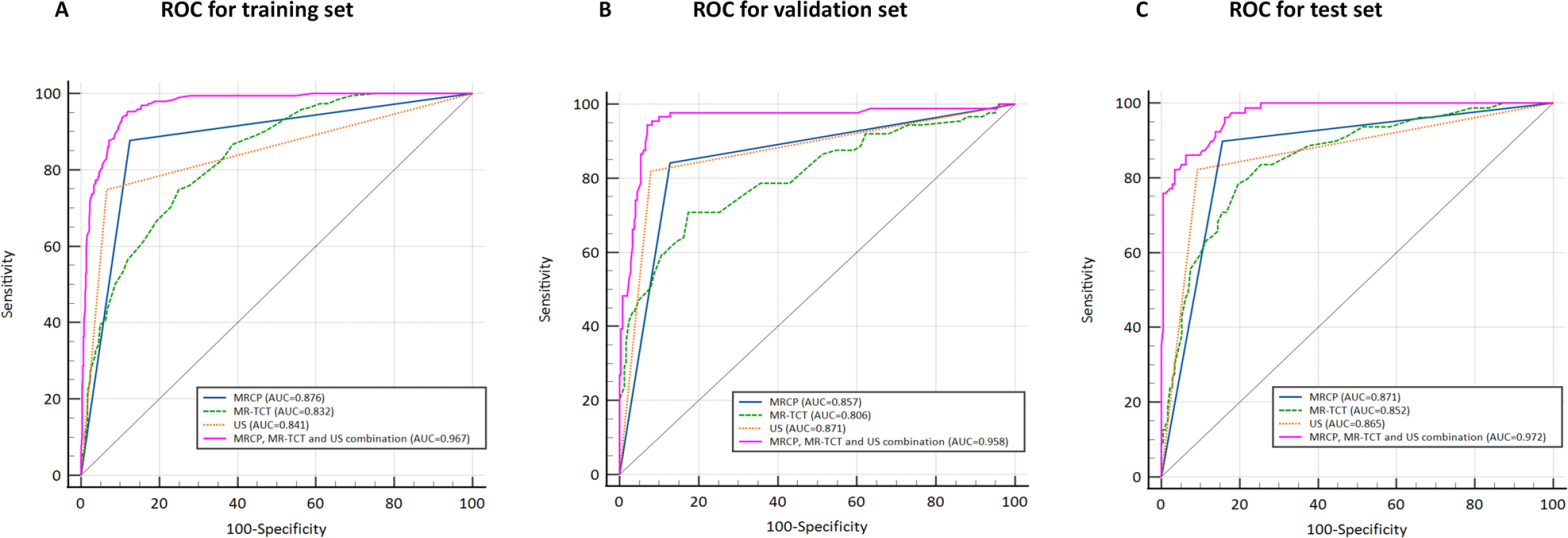
ROC curves of the different models for diagnosing BA. The combination model of 3D MRCP, MR-TCT, and US achieved optimal diagnostic performance, with an AUC of 0.967 (*p* < 0.001) in the training set (**A**), 0.958 (*p* < 0.001) in the validation set (**B**), and 0.972 (*p* < 0.001) in the test set (**C**)

The MR-TCT in the BA group was greater than that in the non-BA group (median [IQR]: 4.40 [3.50, 5.40] vs 3.00 [2.30, 3.60] mm, *p* < 0.001). The optimal cut-off value, determined from all cases via ROC analysis, was 3.75 mm (AUC = 0.828; 95% confidence interval [CI], 0.803 to 0.852; *p* < 0.001). Figure 3 illustrated the ROC curves for BA diagnosis, and AUC values with sensitivity, specificity for each model in the training, validation and test sets were summarized in Table 2. Furthermore, inter-model comparisons were shown in Table 3.

**Table 2:** AUC values of different models

Models	Training set (*n* = 770)				Validation set (*n* = 330)				Test set (*n* = 252)			
	AUC	Sensitivity	Specificity	*p* value	AUC	Sensitivity	Specificity	*p* value	AUC	Sensitivity	Specificity	*p* value
MR-TCT	0.832	74.87%	74.96%	< 0.001	0.806	70.79%	82.57%	< 0.001	0.852	78.48%	80.35%	< 0.001
MRCP	0.876	87.69%	87.48%	< 0.001	0.857	84.27%	87.14%	< 0.001	0.871	89.87%	84.39%	< 0.001
US	0.841	74.87%	93.39%	< 0.001	0.871	82.02%	92.12%	< 0.001	0.865	82.28%	90.75%	< 0.001
MRCP + US	0.931	93.85%	83.65%	< 0.001	0.925	94.38%	83.38%	< 0.001	0.936	96.20%	79.19%	< 0.001
MRCP + MR-TCT	0.948	90.77%	89.74%	< 0.001	0.937	95.51%	84.65%	< 0.001	0.954	87.34%	90.17%	< 0.001
MRCP + MR-TCT + US	0.967	95.38%	88.00%	< 0.001	0.958	94.38%	92.95%	< 0.001	0.972	86.08%	93.64%	< 0.001

MRCP in the model presented the extrahepatic biliary duct (including the right/left hepatic ducts, common hepatic duct, and common bile duct) visualization on MRCP. The diagnosis of BA was established when any segment of the bile duct was non-visualized on MRCP

**Table 3:** Inter-model AUC performance comparisons

Model comparisons	Training set (*n* = 770)			Validation set (*n* = 330)			Test set (*n* = 252)		
	*p* value	95% CI	AUC differences	*p* value	95% CI	AUC differences	*p* value	95% CI	AUC differences
MRCP vs US	0.069	−0.003 to 0.072	0.035	0.619	−0.040 to 0.068	−0.014	0.833	−0.052 to 0.064	0.006
MRCP + US vs US	< 0.001	0.063 to 0.117	0.090	< 0.001	0.047 to 0.118	0.054	< 0.001	0.037 to 0.140	0.071
MRCP + US vs MRCP	< 0.001	0.040 to 0.071	0.055	< 0.001	0.041 to 0.096	0.068	< 0.001	0.039 to 0.092	0.065
MRCP + MR-TCT vs MRCP	< 0.001	0.053 to 0.091	0.072	< 0.001	0.047 to 0.113	0.080	< 0.001	0.053 to 0.112	0.083
MRCP + MR-TCT + US vs MRCP + US	< 0.001	0.021 to 0.052	0.036	0.005	0.010 to 0.055	0.033	0.002	0.013 to 0.057	0.036
MRCP + MR-TCT + US vs MRCP + MR-TCT	< 0.001	0.009 to 0.030	0.019	0.003	0.007 to 0.035	0.021	0.038	0.001 to 0.036	0.018

Specifically, among the 363 infants with surgically confirmed BA, initial US yielded 79 false-negative cases (21.8%), of which 3D MRCP correctly identified 59 (74.7%). Conversely, 3D MRCP yielded 46 false negatives (12.7%), of which US correctly identified 26 (56.5%).

### Comparison of 3D MRCP findings with intraoperative cholangiography and laparoscopic exploration

For the standard of determining the presence of extrahepatic bile ducts, intraoperative laparoscopy and laparoscopic exploration were considered the gold standard in the BA group, while the bile ducts were deemed visible in the non-BA group. In the analysis of 3D MRCP for bile duct visualization, 120 BA cases were excluded owing to the fact that the developmental anatomy of each extrahepatic bile duct segment was not documented during surgical exploration. Table 4 showed the diagnostic accuracy of 3D MRCP for bile duct visualization, and patient details in Supplementary Table S2.

**Table 4:** Diagnostic performance of 3D MRCP for bile ducts visualization

	Right and left hepatic ducts	Common hepatic ducts	Common bile duct
Sensitivity	85.2% (80.5–89.8%)	85.1% (80.4–89.7%)	86.6% (82.2–91.0%)
Specificity	87.5% (85.5–89.6%)	87.7% (85.7–89.9%)	87.8% (85.8–89.8%)
PPV	60.9% (55.6–66.3%)	61.2% (55.8–66.6%)	62.1% (56.8–67.4%)
NPV	96.3% (95.0–97.5%)	96.3% (95.1–97.5%)	96.6% (95.4–97.8%)
Accuracy	87.1% (85.2–89.0%)	87.3% (85.4–89.1%)	87.6% (85.7−89.4%)

Data in parentheses were 95% CI

*PPV* positive predictive value, *NPV* negative predictive value

### The effect of 3D MRCP imaging quality on the diagnostic accuracy of BA

**Fig. 4:**
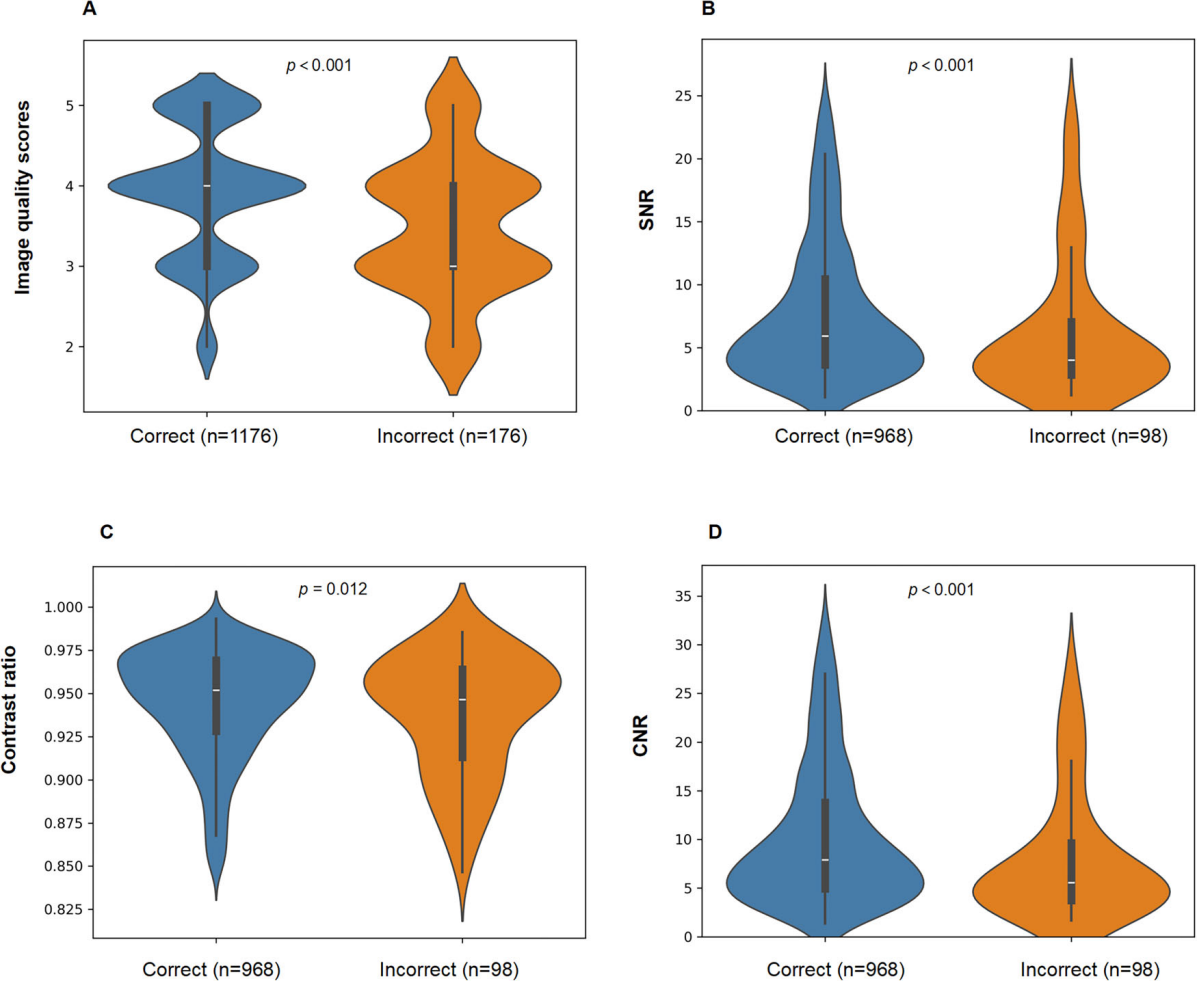
Violin box plots showing image quality scores (**A**), SNR (**B**), contrast ratio (**C**), and CNR (**D**) of 3D MRCP between the correct diagnosis group and the incorrect group. All four image quality parameters were significantly higher in the correct diagnosis group (*p* < 0.05). During the measurements, the GBs of 101 BA patients and 185 non-BA patients were either invisible or too small to measure. As a result, SNR, contrast ratio, and CNR were measured in 262 BA patients and 804 non-BA patients

The correct diagnosis group demonstrated significantly higher image quality scores (median, IQR]: 4.0 [3.0, 5.0] vs 3.0 [3.0, 4.0], *p* < 0.001), SNR (median [IQR]: 6.316 [3.796, 12.251] vs 4.114 [2.844, 8.039], *p* < 0.001), contrast ratio (median [IQR]: 0.950 [0.921, 0.968] vs 0.943 [0.895, 0.962], *p* = 0.012) and CNR (median [IQR]: 8.482 [5.157, 16.264] vs 5.722 [3.732, 10.640], *p* < 0.001) (Fig. 4).

### Intra-and inter-reader consistency evaluation

**Fig. 5:**
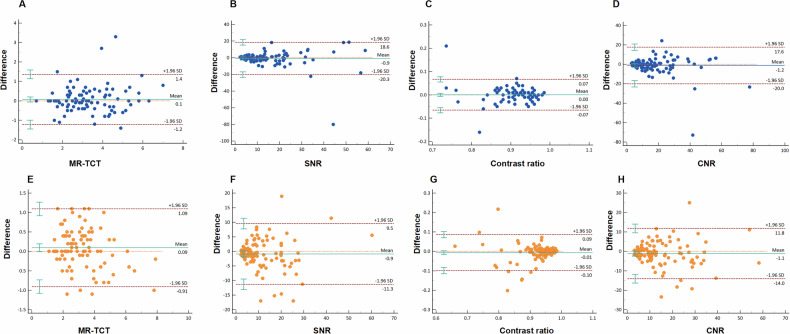
Bland–Altman analysis showed the consistency evaluation of the intra-reader agreements (**A**–**D**) and inter-reader agreements (**E**–**H**) for MR-TCT, SNR, contrast ratio, and CNR of 3D MRCP. The two red horizontal dashed lines demarcating the 95% limits of agreement (LoA), the green solid line indicating the mean difference between measurements, an orange dashed line marking the zero-difference reference level, and the green vertical bars representing the 95% CI of the LoA. More than 95% of the points fell within the mean ± 1.96 SD range (all *p* > 0.05) except for SNR and contrast ratio inter-reader evaluation, where 6% and 7% of the points exceeded LoA, respectively. However, it remained clinically acceptable as 1/6 and 3/7 of the points were observed within the 95% CI of the LoA. Overall, the consistency of the measurements was excellent, with high reproducibility

Intra- and inter-reader agreement was assessed using appropriate statistical methods for each data type: Cohen’s Kappa for categorical duct visualization assessments (right/left hepatic duct, common hepatic duct, and common bile duct) and BA diagnosis on 3D MRCP, intraclass correlation coefficients (ICC) for ordinal image quality scores, and Bland–Altman analysis for continuous variables (MR-TCT, SNR, contrast ratio, and CNR). Both Cohen’s Kappa and ICC values exceeded 0.75, indicating good reliability, and the detailed results were available in Table 5. While the Bland–Altman plots were presented in Fig. 5.

**Table 5:** Intra-and inter-reader agreements for categorical data

	Intra-reader agreement			Inter-reader agreement		
	Correlation	95% CI	*p* value	Correlation	95% CI	*p* value
Image quality score	0.818	0.741–0.873	< 0.001	0.814	0.724–0.875	< 0.001
BA diagnosis on 3D MRCP	0.806	0.661–0.935	< 0.001	0.774	0.627–0.901	< 0.001
Right and left hepatic duct visualization	0.883	0.759–0.973	< 0.001	0.791	0.658–0.913	< 0.001
Common hepatic duct visualization	0.883	0.742–0.976	< 0.001	0.766	0.611–0.886	< 0.001
Common bile duct visualization	0.775	0.606–0.905	< 0.001	0.770	0.639–0.893	< 0.001

*BA* biliary atresia

## Discussion

The study highlighted that 3D MRCP was a highly effective, non-invasive imaging modality that can significantly aid in the diagnosis of BA beyond US. The combination of 3D MRCP, MR-TCT values, and US, substantially improved diagnostic accuracy for BA. The MR-TCT values were significantly higher in the BA group, with a cut-off value of 3.75 mm. Furthermore, the accuracy of BA diagnosis using 3D MRCP depended on achieving higher image quality.

The diagnostic pathway for infants with suspected BA traditionally relies on US as the first-line imaging modality, justified by its accessibility, low cost, and high specificity. However, the inherent limitation of the US lies in its suboptimal sensitivity of 77–82% in the current study, which may result in false-negative findings and consequential delays in intervention. In such clinically ambiguous scenarios where suspicion for BA remains high, our findings advocate for the strategic implementation of 3D MRCP as a decisive adjunct. Despite its greater resource demands, MRCP demonstrated superior diagnostic sensitivity in our cohort, effectively identifying a substantial proportion of BA cases that were initially missed by US.

It is believed that the MR-TCT related to periportal fibrosis has been described in patients with BA and other conditions, and it could serve as a more sensitive and earlier indicator in infants with BA [16, 17]. Our study identified a cutoff of 3.75 mm for distinguishing BA from non-BA, which is lower than the 5.1 mm reported in a previous study by Kim et al [13]. This discrepancy may arise from differences in measurement techniques. While Kim et al measured the MR-TCT at the point of maximum thickness along the right or left main portal vein, our study measured it along the anterior wall of the right portal vein, a method commonly used in most other studies. Additionally, the difference could be influenced by the sample size; our study included a significantly larger sample of 1352 participants, compared to 208 participants in the study by Kim et al. In previous reports that employed the same measurement method as in the current study, such as He et al [18], who reported an MR-TCT with an optimal cutoff value of 4.1 mm in differentiating cystic BA from choledochal cyst. The variations in MR-TCT values can be attributed to the fact that the study distinguishes between different diseases. Additionally, Zhou et al [16] reported a US-TCT cutoff of 2.2 mm in 273 infants with conjugated hyperbilirubinemia, which may be attributed to the differences between the two imaging techniques. MRCP offers superior soft tissue contrast resolution compared to US. It has been suggested that 3D MRCP alone demonstrates good sensitivity and specificity (64% to 100%) for BA, with comparable performance in both preterm and term infants [12, 14]. This aligns with the current study, which shows a sensitivity of 87.3% and specificity of 86.9%. The previous findings indicate that the sensitivity of 3D MRCP is higher than that of US, while its specificity is lower [14], which aligns with the results of this study. More importantly, the results in the study highlighted the significant diagnostic value of combining 3D MRCP and US for the detection of BA. The combined 3D MRCP and US approach demonstrated significantly improved diagnostic performance over either modality alone, leveraging 3D MRCP’s superior visualization of biliary anatomy while complementing US findings for comprehensive BA evaluation. Moreover, the ROC curve emphasized the superior diagnostic performance of the model combining 3D MRCP, MR-TCT, and US. The strength of the combined approach lies in the ability to leverage the complementary advantages of each technique. 3D MRCP offers detailed anatomic visualization of the biliary tree, while MR-TCT is highly sensitive to detecting periportal fibrosis associated with BA [17]. In conclusion, the combination of 3D MRCP, MR-TCT, and US presents a promising diagnostic strategy for BA.

The visibility of the extrahepatic bile ducts has been a critical diagnostic criterion in MRCP-based BA assessments. While Siles et al [19] reported limited common bile duct visualization in only 62.5% (10/16) of healthy neonates and infants, contrasting another study [17] demonstrated 100% detection (9/9) of visible or dilated common bile ducts in non-BA cases. Our study revealed visualization rates of 87.5% for right and left hepatic ducts, 87.7% for common hepatic ducts, and 87.8% for common bile ducts in patients with normal bile ducts. The current study demonstrated superior and more consistent visualization rates compared to previous reports. This enhanced reliability was supported by our substantially larger sample size (*n* = 1352 vs *n* = 9–16 in prior studies), which provided greater statistical power and yielded more precise estimates of the true visualization rates through reduced sampling variability. When evaluated against surgical findings as the reference standard, the PPV of 3D MRCP for bile duct visualization was observed to be relatively low. This finding may be explained by the exclusion of a proportion of BA cases due to the fact that the developmental anatomy of each extrahepatic bile duct segment was not documented during surgical exploration. Despite potential selection biases, the diagnostic utility was affirmed by consistently high performance across other metrics, including sensitivity, specificity, NPV, and accuracy, ranging from 85.1% to 96.6%. These findings suggested that 3D MRCP remained a clinically viable imaging method for assessing extrahepatic bile ducts.

Our findings demonstrated that enhanced MRCP image quality significantly improved diagnostic confidence in identifying BA-characteristic findings. Several factors can influence image quality, including age, higher BMI, inpatient status, use of sedation or general anesthesia, and MR field strength, among others [20–23]. Glenn et al [22] found that using a 1.5-T scanner, in conjunction with respiratory triggering, was associated with a higher likelihood of a successful diagnostic study in children compared to 3-T scanners. This is likely due to fewer artifacts, such as susceptibility and dielectric effect. In contrast, it has been suggested that breath-hold compressed-sensing MRCP provides significantly better perceived image quality at 3-T compared to 1.5-T [24]. In this study, all infants underwent respiratory-triggered or navigator-gated 3D MRCP using either a 3-T or 1.5-T scanner. As is known, 3D MRCP acquisitions can be compromised by suboptimal image quality resulting from artifacts and low SNR [22], a finding that was validated in our study, further emphasizing the importance of identifying factors that influence MRCP image quality in children. The CNR was notably higher in the group with correct diagnoses, indicating better delineation of the bile ducts from surrounding tissues, thereby reducing noise and enhancing contrast. Additionally, contrast ratio was slightly higher in BA patients, underscoring the importance of elevated contrast and spatial resolution in accurately identifying BA-related features, such as malformed bile ducts. The higher SNR further improved bile duct visibility [22], supporting more precise BA diagnoses. Furthermore, inter- and intra-observer agreement for these parameters in all 3D MRCP images was rated as good, suggesting that the results were reliably reproducible.

The study has several limitations. First, it involved multiple MRI scanner manufacturers, which introduced potential confounding factors when assessing image quality and diagnostic accuracy. However, we did not conduct a more detailed analysis to account for these factors.

Second, only subjects with both MR and US were included, excluding those with only US. To some extent, this limited the comprehensive assessment of ultrasound’s standalone ability to exclude BA. Third, the US examination results used for comparison were based on the reports and archived images at the time, without consistent validation of their accuracy. Fourth, cases with very poor image quality were excluded from the study. While such cases represent a small proportion in clinical practice, their exclusion might have resulted in an overestimation of 3D MRCP for BA in our study.

## Conclusions

In conclusion, 3D MRCP serves as a critical adjunct to US in the diagnosis of suspected BA, primarily by enabling the identification of specific imaging features—the non-visualization of the extrahepatic bile duct and an MR-TCT > 3.75 mm. Given its principal advantage in sensitivity and direct multi-planar visualization, 3D MRCP is imperative for cases with high clinical suspicion of BA but inconclusive US findings, enabling accurate diagnosis, reduced false negatives, and timely intervention.

## Supplementary information

ELECTRONIC SUPPLEMENTARY MATERIAL

## Data Availability

The datasets analyzed in this study are subject to institutional data-sharing restrictions and thus cannot be made publicly accessible. However, de-identified data may be made available upon formal request to the corresponding author, pending institutional review and approval of the proposed use.

## Abbreviations

3D-MRCP: Three-dimensional MRCP
AUC: Area under the ROC curve
BA: Biliary atresia
CI: Confidence interval
CNR: Contrast-to-noise ratio
GB: Gallbladder
ICC: Intraclass correlation coefficient
MRCP: Magnetic resonance cholangiopancreatography
MR-TCT: MR triangular cord thickness
NPV: Negative predictive value
PPV: Positive predictive value
ROC: Receiver operating characteristic curve
SD: Standard deviation
SI: Signal intensity
SNR: Signal-to-noise ratio
US: Ultrasound
