# Supplementary material for: Adjunctive value of 3D MRCP in biliary atresia: a retrospective two-center analysis of cholestatic infants
Source: Insights Imaging. 2025 Dec 17;16:277. doi: 10.1186/s13244-025-02165-5 (PMC12712274; doi:10.1186/s13244-025-02165-5)
Supplement: Supplementary file 1 — ELECTRONIC SUPPLEMENTARY MATERIAL [file 13244_2025_2165_MOESM1_ESM.pdf]

Adjunctive value of 3D MRCP in biliary atresia: A Retrospective two-center analysis of cholestatic infants

ELECTRONIC SUPPLEMENTARY MATERIAL

Supplementary Table S1. Detailed parameters of MR scanners

MR systems	Tesla (T)	Scanning sequences	TR/TE (msec/msec)	Slice thickness (mm)	Slice gap (mm)	FOV (mm×mm)	Matrix
Siemens Skyra	3.0	Axial T1-weighted	4.0/1.3	2	0	210 × 320	256 × 224
		Axial T2-weighted	3339.5/83	3	0.6	230 × 230	320 × 320
		Coronal 3D MRCP	5357.7/900	1	0	280 × 280	320 × 320
Siemens Prisma	3.0	Axial T1-weighted	3.5/1.7	2	0	300 × 300	256 × 205
		Axial T2-weighted	3908.3/87	3	0.6	280 × 280	256 × 256
		Coronal 3D MRCP	5969.1/409	1	0	320 × 320	384 × 346
Philips Achieva	1.5	Axial T1-weighted	10/4.6	5	0.6	240 × 240	184 × 184
		Axial T2-weighted	2624/73	5	0.6	250 × 250	288 × 288
		Coronal 3D MRCP	2132/400	2	0	260 × 238	324 × 297

Supplementary Table S2. Number of cases diagnosed via 3D MRCP compared with reference standard for bile ducts visualization

		Reference standard		
		Positive	Negative	Total
Right and left hepatic ducts	Positive	195	125	320
	Negative	34	878	912
	Total	229	1003	1232
Common hepatic ducts	Positive	194	123	317
	Negative	34	881	915
	Total	228	1004	1232
Common bile duct	Positive	200	122	322
	Negative	31	879	910
	Total	231	1001	1232
